# Plasma-Derived Exosomal hsa-miR-4488 and hsa-miR-1228-5p: Novel Biomarkers for Dermatomyositis-Associated Interstitial Lung Disease with Anti-Melanoma Differentiation-Associated Protein 5 Antibody-Positive Subset

**DOI:** 10.1155/2021/6676107

**Published:** 2021-07-28

**Authors:** Danli Zhong, Chanyuan Wu, Dong Xu, Jingjing Bai, Qian Wang, Xiaofeng Zeng

**Affiliations:** Department of Rheumatology, Peking Union Medical College Hospital, Peking Union Medical College & Chinese Academy of Medical Sciences, National Clinical Research Center for Dermatologic and Immunologic Diseases, Key Laboratory of Rheumatology and Clinical Immunology, Ministry of Education, Beijing 100730, China

## Abstract

The present study is aimed at profiling circulating exosome-derived microRNAs (miRNAs/miRs) from patients with dermatomyositis (DM), in particular those complicated with interstitial lung disease (ILD) with anti-melanoma differentiation-associated protein 5 (MDA5) antibody-positive. Fifteen participants were enrolled, including five patients with DM complicated with ILDs prior to treatment with circulating anti-MDA5 antibody-positive status [DM-ILD-MDA5 Ab(+)], five DM patients without ILDs who were negative for 16 detectable myositis-specific antibodies [DM-nonILD-MSA16(-)], and five age- and gender-matched healthy donor controls (HCs). The characteristics of the exosomes extracted by Ribo™ Exosome Isolation Reagent were identified using transmission electron microscopy (TEM), nanoparticle tracking analysis (NTA), and flow cytometry. Differentially expressed miRNAs, determined by next-generation deep sequencing, were identified through the criteria of ∣log2 fold change | ≥1 and *P* < 0.01. A total of 38 miRNAs were significantly upregulated in exosomes from patients with DM-ILD-MDA5 Ab(+) compared to those from HC, while 21 miRNAs were significantly downregulated. Compared to exosomes derived from patients with DM-nonILD-MSA16(-), 51 miRNAs were significantly upregulated and 33 miRNAs were significantly downregulated from patients with DM-ILD-MDA5 Ab(+). A total of 73 exosomal miRNAs were significantly differentially expressed between DM-nonILD-MSA16(-) and HC. In particular, two miRNAs, *Homo sapiens*- (hsa-) miR-4488 and hsa-miR-1228-5p, were common differentially expressed miRNAs among three comparisons. GO and KEGG analyses suggested that several pathways may contribute the pathogenesis of DM-ILD-MDA5 Ab(+) and DM-nonILD-MSA16(-), while PPI network analysis of hsa-miR-4488 and hsa-miR-1228-5p indicated that their predicted target genes, DExD-box helicase 39B and MDM2, may be involved in the mechanisms of DM-ILD-MDA5 Ab(+).

## 1. Introduction

Exosomes, small vesicles (30-150 nm in diameter) of endocytic origin, are formed in endosomal multivesicular compartments and are secreted into the extracellular space when these compartments fuse with the plasma membrane. Exosomes may be purified from cell cultures and body fluids, including plasma/serum, saliva, urine, and cerebrospinal fluid. Exosomes contain various functional small RNAs, mRNAs, and functional proteins, thereby mediating long distant cell-cell communication. MicroRNAs (miRNAs/miRs) are small noncoding RNAs that posttranscriptionally regulate target genes by base-pairing with 3′-untranslated regions of target mRNA [1]. It has been reported that exosomal miRNAs are extra stable in various storage conditions [2] and are protected from RNase treatment, thereby providing a protected and enriched source of miRNA [3]. These results suggest that exosome-derived miRNA (exRNA) has the potential for diagnosis, treatment, and prognosis prediction as a stable biomarker, and it overlaps with the even newer field of exRNA-mediated communication.

Dermatomyositis (DM) encompasses a spectrum of rare, heterogeneous autoimmune diseases characterized by proximal symmetric muscle weakness, skin rash, multiple organ involvement, and paraneoplastic syndromes [4]. The etiology of DM is unknown; however, cross-talk between genetic and environmental factors contributes to the pathology [5]. Over the past decades, new subsets of DM have become increasingly recognized. Patients with DM may be subclassified on the basis of clinical and laboratory features, among which myositis-specific autoantibodies (MSAs) have a crucial role in serologically defined subsets of DM [6]. When autoantibodies to a 140 kDa protein were first discovered in 2005 in patients with clinically amyopathic DM [7] who predominately displayed cutaneous manifestations for abnormally long periods of time (≥6 months) without accompanying muscle weakness [8], subsequent evidence demonstrated that melanoma differentiation-associated gene-5 (MDA5) was the target autoantigen [9]. MDA5, a crucial member of the retinoic acid-inducible gene-like receptor family, recognizes viral double-strand RNA with the production of type I interferon, thereby protecting the host against invasion of virus [10]. Patients with anti-MDA5 autoantibodies (anti-MDA5 Ab) are at risk of an acute, rapidly progressive, and potentially fatal form of interstitial lung disease (ILD) that requires aggressive systemic immunosuppressive therapy and is associated with high morbidity and mortality rates [11, 12]. It has been reported that the prevalence of ILD in DM with anti-MDA5 Ab-positive status was higher in Asian populations than that in Europeans [13–15]. The absence of anti-MDA5 Ab in other connective tissue disorders and the presence of anti-MDA5 Ab being mutually exclusive of other MSAs in patients with DM suggested that a specific immune response may govern this subtype of DM. However, the underlying mechanisms of DM-associated ILD with anti-MDA5 Ab remain elusive. As the lack of homogeneous grouping based on the antibody profiles perturbs the diagnostic, therapeutic, and prognostic stratification of these patients, exosome-derived miRNAs of circulating plasma were profiled in the present study using next-generation sequencing (NGS) from two subsets of DM patients: patients with DM complicated with ILDs prior to treatment with circulating anti-MDA5 Ab-positive status [DM-ILD-MDA5 Ab(+)] and patients without ILDs who were negative for 16 detectable MSA [DM-nonILD-MSA16(-)]. Patients were considered to be DM-nonILD-MSA16(-) when 16 MSAs were undetected in serum, including anti-aminoacyl-transfer (t)RNA synthetase autoantibodies (anti-Jo-1, anti-PL-7, anti-PL-12, anti-EJ, and anti-OJ), anti-Ro-52, anti-signal recognition particle antibodies (anti-SRP), anti-PM-SCL75, anti-PM-SCL100, anti-KU, anti-small ubiquitin-like modifier activating enzyme antibodies (anti-SAE), anti-nuclear matrix protein 2 antibodies (anti-NXP2), anti-MDA5, transcriptional intermediary factor-1*γ* autoantibodies (anti-TIF1*γ*), anti-MI-2*α*, and anti-MI-2*β* antibodies.

## 2. Materials and Methods

### 2.1. Characteristics of Eligible Patients

A total of 10 patients diagnosed with DM and five healthy volunteer controls (HCs) were included in the present study from Peking Union Medical College Hospital (PUMCH). The individuals in control group (age- and sex-matched) did not suffer autoimmune or inflammatory diseases. The consecutive patients were enrolled from the Department of Rheumatology, Peking Union Medical College Hospital affiliated to Peking Union Medical College & Chinese Academy of Medical Sciences (Beijing, China) between November 1^st^, 2017 and January 1^st^, 2018. All patients and controls provided written informed consent according to the Declaration of Helsinki. This study was approved by the Ethics Committee of Peking Union Medical College Hospital (Beijing, China; no. S-478).

The diagnosis of DM was determined according to the Bohan and Peter criteria [16]. ILD was considered if patients fulfilled the following criteria: (i) radiological signs on high-resolution computerized tomography and (ii) impaired pulmonary function (total lung capacity, diffusion capacity for carbon monoxide of the lung < 80% of predicted and forced vital capacity < 80% of predicted).

Serum MSA profiles including anti-aminoacyl-tRNA synthetase autoantibodies (anti-Jo-1, anti-PL-7, anti-PL-12, anti-EJ, and anti-OJ), anti-RO-52, anti-SRP, anti-PM-SCL75, anti-PM-SCL100, anti-KU, anti-SAE, anti-NXP2, anti-MDA5, anti-TIF1*γ*, anti-MI-2*β*, and anti-MI-2*α* were measured using commercial kits by line blot assays (Myositis Profile Euroline kit; Euroimmun AG) [17]. Patients with DM were divided into two groups according to lung involvement and the presence of MDA5 autoantibodies. A total of 5 patients diagnosed as DM-ILD-MDA5 Ab(+) were eligible and naive to treatment with prednisone or immunosuppressant drugs prior to sample collection. None of the anti-MDA5 Ab(+) DM patients had MSAs other than anti-MDA5 antibody. The other five patients diagnosed with DM without ILD were negative for serum MSAs. The characteristics of the patients included are summarized in Table 1. Patients with malignancy, pulmonary infection, or overlapping connective tissue diseases were excluded.

### 2.2. Blood Collection and Isolation of Plasma Exosomes

A total of 20 ml of whole blood were collected from the cubital vein of patients using blood collection tubes containing K_2_EDTA. Blood samples were centrifuged at 4,000 × g at 4°C for 5 min using a swinging bucket rotor within 4 h of the blood draw. The upper supernatant (plasma) was carefully transferred to a new tube with a conical bottom without disturbing the intermediate buffy coat layer and was stored at -80°C prior to the isolation of plasma exosomes.

Plasma exosomes were extracted using a commercial kit (Ribo™ Exosome Isolation Reagent; RiboBio) following the manufacturer's protocol. In brief, plasma samples were thawed from -80°C and were centrifuged at 2,000 × g for 20 min at 4°C to remove cells and cell debris. Subsequently, the supernatants were transferred to sterile polyethylene tubes with addition of 1/3 volume of Ribo™ Exosome Isolation Reagent (RiboBio). The samples were incubated at 4°C for 30 min. Subsequently, the samples were centrifuged at 15,000 × g at 4°C for 2 min to obtain white pellets (exosomes) following removal of the upper supernatant. The isolated exosome samples were stored at -80°C for the subsequent assays. The study was designed to be consistent with the minimal experimental requirement for extracellular vesicles from the statement from the International Society for Extracellular Vesicles [18].

### 2.3. Identifications of Exosomes Using TEM

Biological transmission electron microscopy (TEM) was performed to determine the characteristics of exosomes. Exosomes were deposited onto a copper grid for 5 min at room temperature. Subsequently, the grid was negatively stained with 2% uranyl acetate for 5 min. The grid was then imaged under a biological TEM (Tecnai™ G^2^ spirit BioTWIN; FEI Co.) with an accelerating voltage set to 120 kV.

### 2.4. Nanoparticle Tracking Analysis (NTA)

Quantification and size determination of plasma exosomes were performed using a NanoSight NS300 instrument (Malvern Instruments, Inc., UK) equipped with the NTA 3.0 software (Malvern Instruments, Inc.). In brief, exosomes were diluted with deionized water by 1 : 10,000, which provided particle concentrations of ~5 × 10^7^/ml prior to examination using the NTA system. Exosome samples were then injected into a NanoSight sample cubicle (Malvern Instruments, Inc., UK) by a syringe. A total of 5 recordings of 30 sec each were performed, and data from at least 5,000 individual particle tracks per sample were analyzed. The data of exosomal size distribution and mean number of particles per milliliter were expressed as the mean ± standard deviation.

### 2.5. Flow Cytometry

Plasma exosomes isolated with the commercial kits were analyzed using a BD Accuri C6 flow cytometer (BD Biosciences) with the FlowJo software 10.0.7 (FlowJo LLC). Exosomes were stained by CD63-FITC (BD Biosciences; cat. no. 557288) and CD81-FITC (BD Biosciences; cat. no. 551108) for 20 min at 4°C, both at a dilution of 1 : 500. In brief, exosomes were resuspended in 100 *μ*l PBS that had been filtered using 0.22 *μ*m filters. The samples were incubated with FITC-conjugated CD63 or CD81 antibody in PBS for 15-20 min at 4°C with gentle agitation. After washing in PBS, the samples were resuspended in 100 *μ*l PBS and analyzed using flow cytometry.

### 2.6. Exosomal RNA Extraction, Library Preparation, and NGS

Total RNA was extracted from isolated exosomes using TRIzol reagent (Invitrogen; Thermo Fisher Scientific, Inc.) according to the manufacturer's protocol. The purity and concentration of RNA was assessed using the Nanodrop-1000 (Thermo Fisher Scientific, Inc.). Each RNA sample had an absorbance at 260 nm (A260)/A280 ratio > 1.8 and an A260/A230 ratio of >2.0. RNA integrity was evaluated using the Agilent 2200 TapeStation (Agilent Technologies, Inc.), and each sample had an RNA integrity number (RIN) > 7.0. In brief, RNAs from exosomes were ligated with 3′RNA adapter and followed by 5′adapter ligation. Subsequently, the adapter-ligated RNAs were subjected to reverse transcription PCR and amplified with a low-cycle as previously described [19]. Then, PCR products were size-selected using PAGE according to the instructions of the NEBNext® Multiplex Small RNA Library Prep Set for Illumina® (New England Biolabs, Inc.). The purified library products were evaluated using the Agilent 2200 TapeStation and were diluted to 10 pM for cluster generation *in situ* on the HiSeq2500 single-end flow cell followed by sequencing (1 × 50 bp) on the HiSeq 2500 system.

### 2.7. Quality Control and Mapping to the Reference Genome

The raw reads were preprocessed to trim 3′ and 5′ adapters, and the low-quality reads were removed to obtain clean reads. The GC content, Q20, and Q30 of the clean data were also calculated. miRBase (version 21.0; http://www.mirbase.org) was used to build the index of the reference genome for the components and expression profiles of all types of small RNAs [20]. The miRNA expression levels were estimated as the number of reads per million (RPM) using the following formula:

(1) $$\begin{array}{c} \text{RPM}=\frac{\text{number of reads mapping to miRNA}}{\text{number of reads in clean data}}\times 106. \end{array}$$

### 2.8. Differentially Expressed (DE) miRNA Analysis

The edgeR package in Bioconductor was used to analyze DE miRNAs between two groups [21]. DE miRNAs were defined as those fulfilling the criteria of ∣log2 (fold change) | ≥1 and *P* < 0.01. Due to the low abundance of small noncoding RNAs in exosomes, the number of DE miRNAs based on adjusted *P* values was too low to perform any further analysis. In fact, thresholds in high-throughput sequencing may be set at unadjusted *P* values to determine the DE miRNAs, which has been reported previously [22]. If the expression level was too low to detect (displayed as zero), the expression level was set as 0.001 in order to calculate the fold change.

The R package multiMiR (http://multimir.ucdenver.edu/) was used to predict candidate target genes (CTGs) of miRNA. This database compiles 14 existing miRNA-target prediction programs from experimental, computational, drug, and disease resources, including DIANA-microT, EIMMo, MicroCosm, miRanda, miRDB, PicTar, PITA, and Targetscan [23].

### 2.9. Functional Enrichment Analysis

To explore the potential function of miRNAs, Gene Ontology (GO) functional annotation and Kyoto Encyclopedia of Genes and Genomes (KEGG; http://www.genome.jp/) enrichment analyses were performed on predicted target genes of differentially expressed miRNA using the clusterProfiler package [24]. GO functional analysis consists of the category biological process (BP), cellular component (CC), and molecular function (MF). The results of the analysis were extracted under the threshold of an adjusted *P* value <0.05.

### 2.10. Construction of miRNA-mRNA Regulatory Network and Protein-Protein Interaction (PPI) Network Analysis

To establish the miRNA-mRNA network, the analysis was focused on the DE miRNAs among three comparisons. Cytoscape was used to draw the network for visualization. Subsequently, the DE miRNAs were further analyzed using the Search Tool for the Retrieval of Interacting Genes and Proteins (STRING) database (http://string-db.org) to determine the interactive relationships of CTGs. Based on the results obtained with STRING, the PPI network was visualized utilizing the Cytoscape software (version 3.7.1) [25].

### 2.11. Statistical Analysis

Demographic data were expressed as the mean ± standard deviation, while differences between two groups were assessed by the unpaired *t*-test as appropriate. *P* values for differential analysis were two-sided, and *P* < 0.01 was considered to indicate statistical significance. All statistical analyses were performed using R packages for R 3.5.1 or GraphPad Prism 7.0 (GraphPad Software, Inc.).

## 3. Results

### 3.1. Characterization of Plasma Exosomes

**Figure 1:**
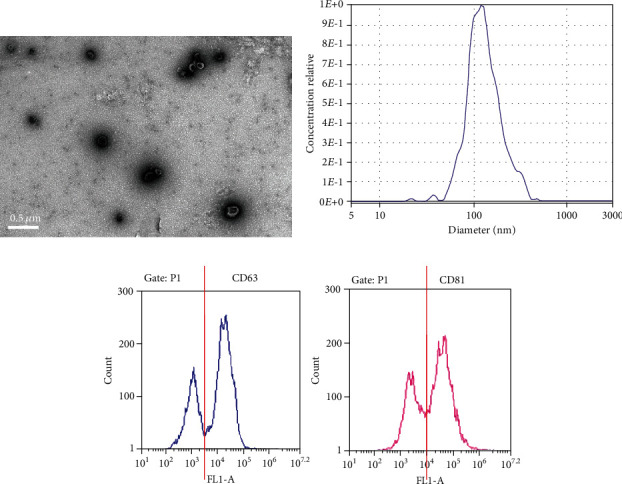
Characteristics of plasma exosomes. (a) Representative transmission electron microscopy image of plasma exosomes (scale bar, 0.5 *μ*m). (b) Representative results of size distribution of exosomes analyzed by nanoparticle tracking analysis. (c) Representative flow cytometric histograms for the determination of the cell surface markers on plasma-derived exosomes.

A total of ten patients and five healthy controls were recruited in the present study. The basic demographic characteristics of enrolled individuals are presented in Table 1. There were no significant differences in the sex and age distributions among these three groups. Plasma-derived exosomes were characterized by TEM, NTA, and flow cytometry. The morphology and ultrastructure of the exosomes was assessed using TEM technology, and the characteristic round- or oval-shaped bodies were visualized. The NTA results indicated that exosomes obtained with the commercial kit were 121.9 ± 64.8 nm in size with a concentration of ~5 × 10^12^ particles/ml, which was expected according to the kit manual. Surface tetraspanins on exosomes, CD63 and CD81, were confirmed using flow cytometry (Figure 1). These results suggested that the commercial kits for exosome separation are reliable and generated acceptable yields as expected.

### 3.2. Classification of Circulating Exosomal Small RNAs

**Figure 2:**
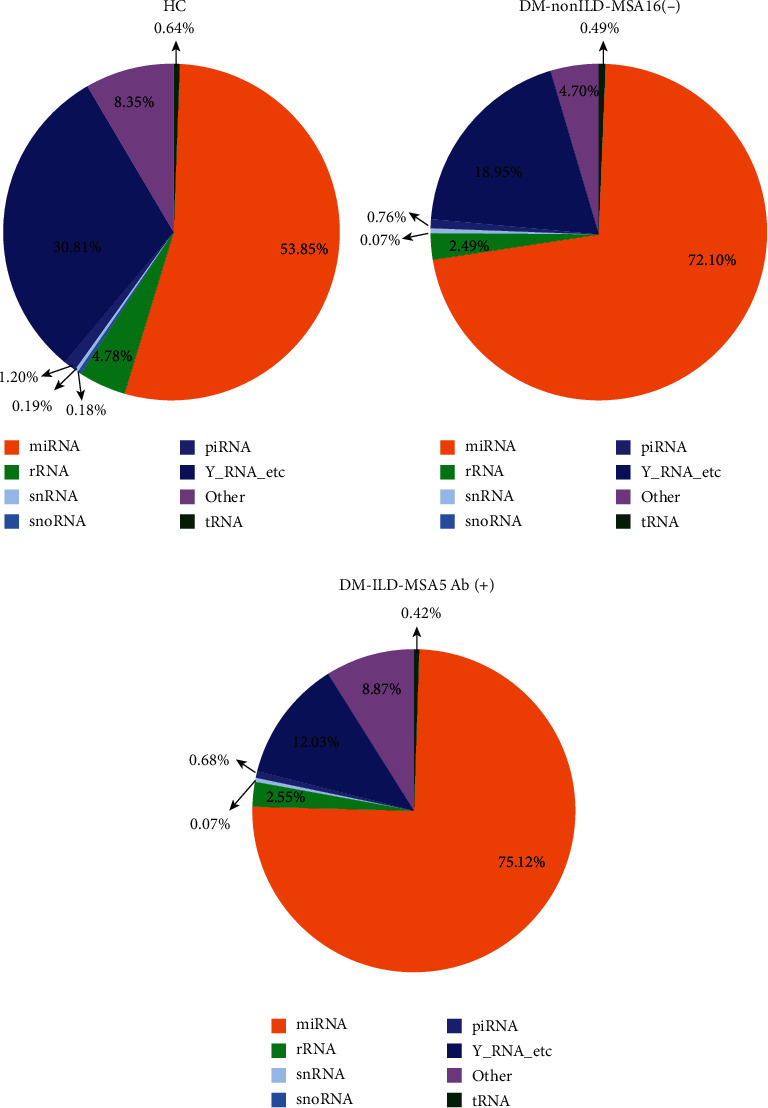
Pie charts displaying the distribution of different classes of small RNAs derived from exosomes in (a) HC, (b) DM-nonILD-MSA16(-), and (c) DM-ILD-MDA5 Ab(+). HCs: healthy controls; DM-ILD-MDA5 Ab(+): DM patients with ILDs and anti-melanoma differentiation-associated protein 5 antibody-positive status; DM-nonILD-MSA16(-): DM patients without ILDs who were negative for 16 detectable myositis-specific antibodies; ILD: interstitial lung disease; DM: dermatomyositis; miRNA: microRNA; rRNA: ribosomal RNA; snRNA: small nuclear RNA; snoRNA: small nucleolar RNA; piRNA: Piwi-interacting RNA; tRNA: transfer RNA.

Exosomes have been demonstrated to encompass various types of small RNAs, including miRNA, tRNA, ribosomal (r)RNA, small nuclear (sn)RNA, small nucleolar (sno)RNA, Piwi-interacting (pi)RNA, Y_RNA, and unannotated RNAs [26, 27]. Therefore, the exosomal small RNAs isolated from patients of the three groups were classified by mapping the clean reads to a noncoding RNA database, including miRBase version 21.0 (miRNA database; http://www.mirbase.org), Rfam 12.1 (tRNA, rRNA, snRNA, and snoRNA databases; http://rfam.xfm.org), and piRNAbank (piRNA database; http://pirnabank.ibab.ac.in). The proportion of miRNA in the total small RNA isolated from exosomes in DM-ILD-MDA5 Ab(+), DM-nonILD-MSA16(-), and HC was 75.12, 72.10, and 53.85%, respectively (Figure 2). Using miRBase version 21.0 as a reference, 912, 895, and 876 known miRNAs were identified in DM-ILD-MDA5 Ab(+), DM-nonILD-MSA16(-), and HC plasma exosomes, respectively.

### 3.3. DE miRNA

**Figure 3:**
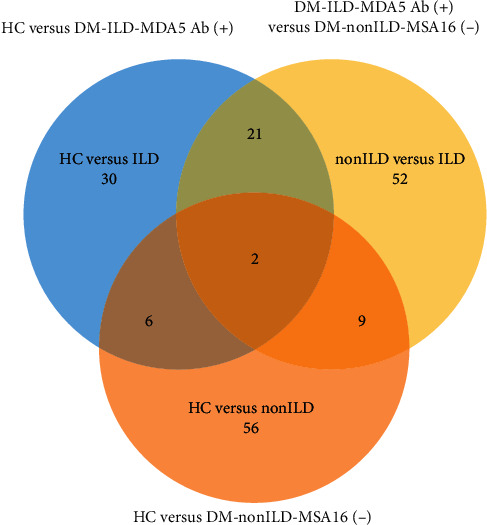
Comparison between identified miRNAs released from exosomes of DM-ILD-MDA5 Ab(+), DM-nonILD-MSA16(-), and HC. Venn diagram demonstrating that the homologous exosomes from three comparisons have two common miRNAs (hsa-miR-1228-5p and hsa-miR-4488). HC: healthy controls; DM-ILD-MDA5 Ab(+): DM patients with ILDs and anti-melanoma differentiation-associated protein 5 antibody-positive status; DM-nonILD-MSA16(-): DM patients without ILDs who were negative for 16 detectable myositis-specific antibodies; ILD: interstitial lung disease; DM: dermatomyositis; miRNA/miR: microRNA; hsa: *Homo sapiens*.

**Figure 4:**
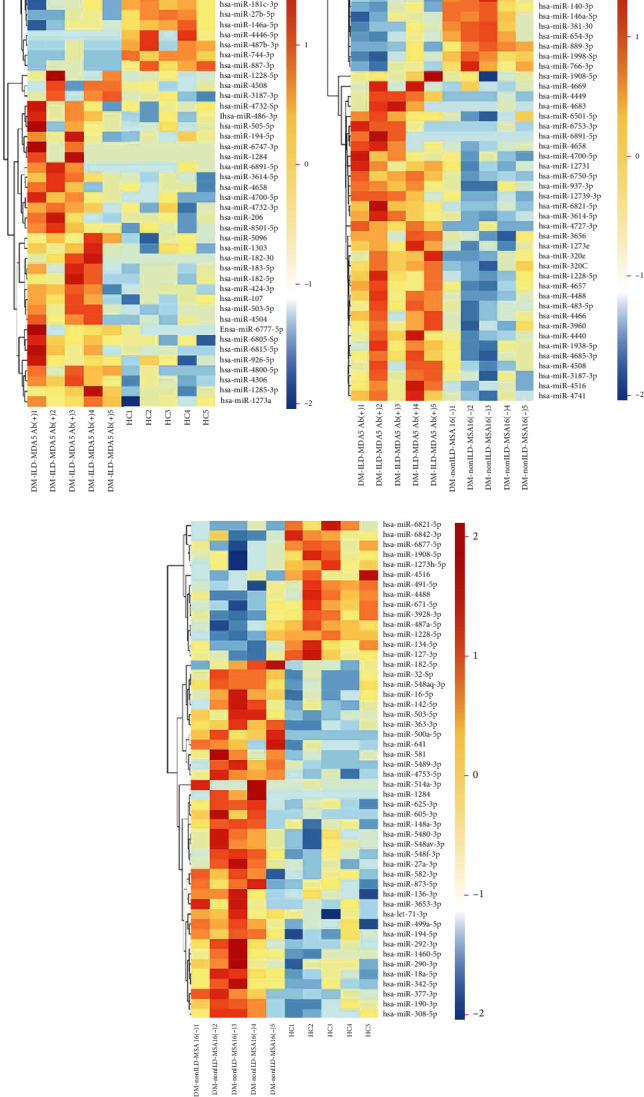
Heatmaps of the top 50 significantly differentially expressed miRNAs between pairs of groups. (a) HC vs. DM-ILD-MDA5 Ab (+), (b) DM-nonILD-MSA16 (-) vs. DM-ILD-MDA5 Ab(+), and (c) HC vs. DM-nonILD-MSA16(-). Gene expression data were obtained using next-generation sequencing on the Illumina HiSeq 2500 platform. The miRNAs are displayed on the vertical axis and the different numbered subjects on the horizontal axis. HCs: healthy controls; DM-ILD-MDA5 Ab(+): DM patients with ILDs and anti-melanoma differentiation-associated protein 5 antibody-positive status; DM-nonILD-MSA16(-): DM patients without ILDs who were negative for 16 detectable myositis-specific antibodies; ILD: interstitial lung disease; DM: dermatomyositis; miRNA/miR: microRNA; hsa: *Homo sapiens*.

After filtering out miRNAs with low expression (RPM < 10 in circulating exosomes), using ∣log2 fold change | ≥1 and *P* < 0.01 as a cut-off threshold, numerous miRNAs were indicated to be significantly differentially expressed between two groups. Among the DE miRNAs, 38 miRNAs were upregulated and 21 were downregulated in DM-ILD-MDA5 Ab(+) exosomes as compared to HC exosomes. Compared to patients with DM-nonILD-MSA16(-), 51 miRNAs were significantly upregulated and 33 miRNAs were significantly downregulated in patients with DM-ILD-MDA5 Ab(+). A total of 73 miRNAs were significantly differentially expressed between DM-nonILD-MSA16(-) and HC, including 50 upregulated and 23 downregulated miRNAs in patients with DM-nonILD-MSA16(-) vs. HC. A Venn diagram demonstrated that exosomal miRNA profiles from DM-ILD-MDA5 Ab(+), DM-nonILD-MSA16(-), and HC had two common miRNAs [*Homo sapiens*- (hsa-) miR-4488 and hsa-miR-1228-5p; Figure 3]. In the present study, hsa-miR-1228-5p was upregulated in the DM-ILD-MDA5 Ab(+) group (fold change = 2.1956, *P* = 0.0034) and downregulated in the DM-nonILD-MSA16(-) group (fold change = −3.4834, *P* = 0.0034) when compared with the HC group, while hsa-miR-4488 was upregulated in the DM-ILD-MDA5 Ab(+) group as compared with the DM-nonILD-MSA16(-) group (fold change = 17.7211, *P* = 7.79 × 10^−7^) and the HC group (fold change = 2.4375, *P* = 0.0099). The DE miRNAs are displayed in Tables SI-III. Heatmaps revealed that the top 50 DE miRNAs had high intragroup similarity and low intergroup similarity (Figure 4).

### 3.4. Functional Enrichment of Top 10 DE miRNA-Targeted Genes

**Figure 5:**
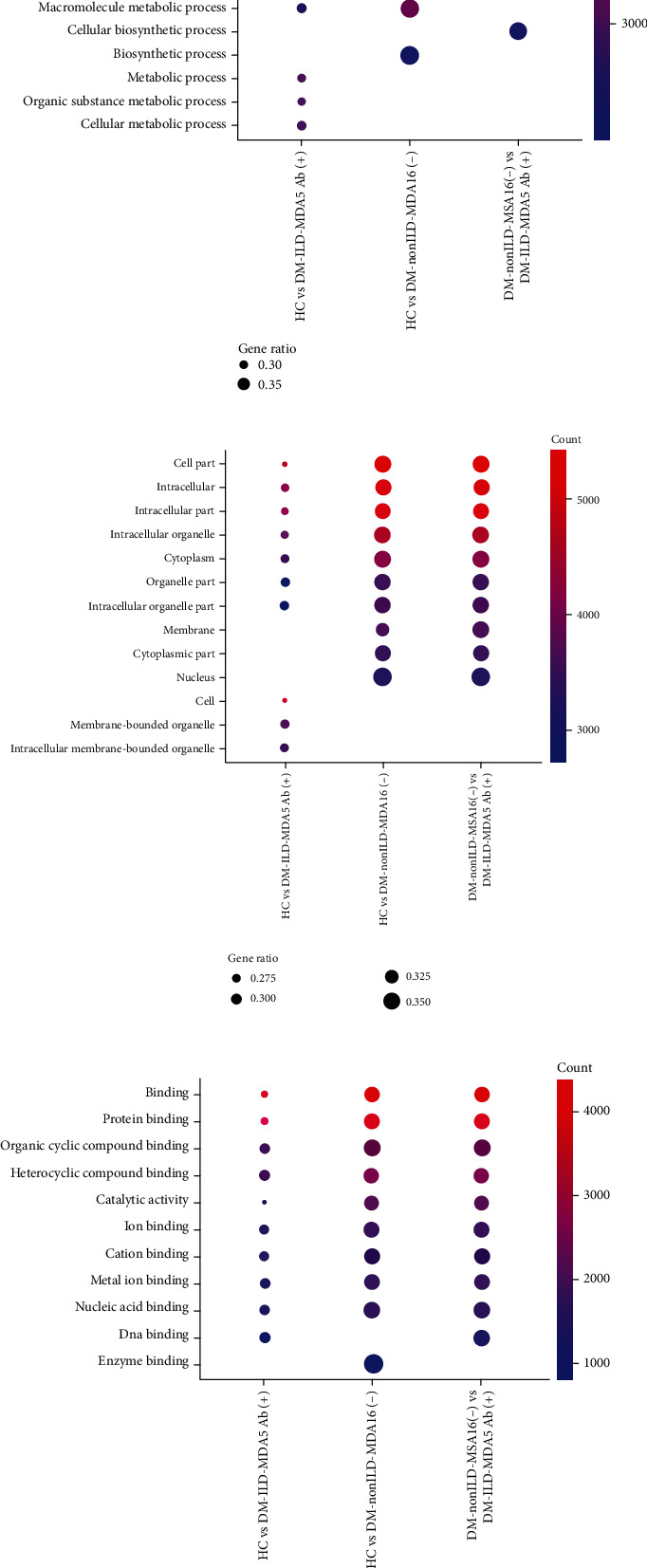
Top 10 Gene Ontology terms in the categories of (a) biological process, (b) cellular component, and (c) molecular function regulated by candidate target genes of significantly differentially expressed microRNAs in plasma exosomes. HC: healthy controls; DM-ILD-MDA5 Ab(+): DM patients with ILDs and anti-melanoma differentiation-associated protein 5 antibody-positive status; DM-nonILD-MSA16(-): DM patients without ILDs who were negative for 16 detectable myositis-specific antibodies; ILD: interstitial lung disease; DM: dermatomyositis.

**Figure 6:**
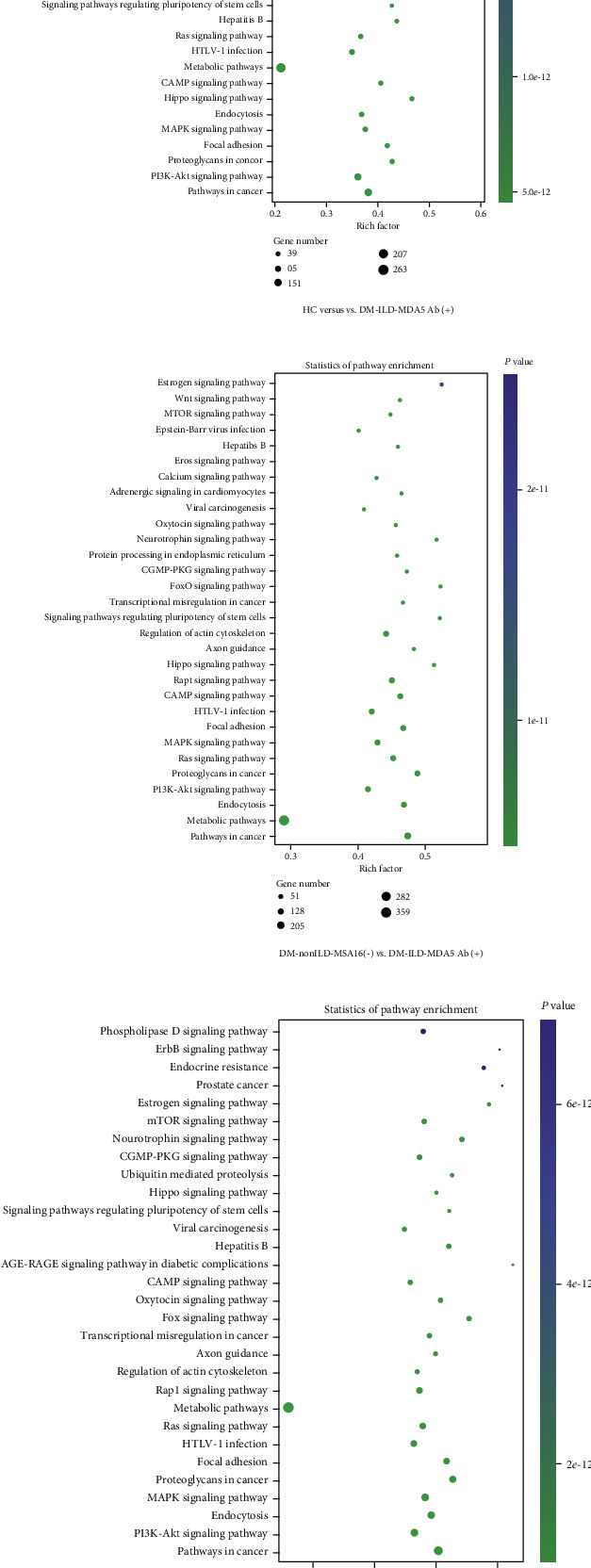
Top Kyoto Encyclopedia of Genes and Genomes pathways regulated by candidate target genes of significantly differentially expressed microRNAs. (a) HC vs. DM-ILD-MDA5 Ab(+). (b) DM-nonILD-MSA16(-) vs. DM-ILD-MDA5 Ab(+). (c) HC vs. DM-nonILD-MSA16(-). HCs: healthy controls; DM-ILD-MDA5 Ab(+): DM patients with ILDs and anti-melanoma differentiation-associated protein 5 antibody-positive status; DM-nonILD-MSA16(-): DM patients without ILDs who were negative for 16 detectable myositis-specific antibodies; ILD: interstitial lung disease; DM: dermatomyositis; PKG: protein kinase G; Fox: forkhead box; cGMP: cyclic guanosine monophosphate; cAMP: cyclic adenosine monophosphate; HTLV: human T-lymphotropic virus.

Figure 5 presents the top 10 GO terms in the categories of BP (Figure 5(a)), CC (Figure 5(b)), and MF (Figure 5(c)) for CTGs of DE miRNAs, indicating certain similarities and differences among the various subsets when considering the different control groups. In terms of MF, CTGs of DE miRNAs in DM-ILD-MDA5 Ab(+) were substantially associated with protein binding, DNA binding, catalytic activity, organic cyclic compound binding, ion binding, cation binding, metal ion binding, nucleic acid binding, and heterocyclic compound binding when compared to HC and DM-nonILD-MSA16(-). In comparison to HC, CTGs of DE miRNAs in DM-nonILD-MSA16(-) were enriched in enzyme binding rather than DNA binding. Regarding the category of CC, the top 10 terms in DM-nonILD-MSA16(-) vs. DM-ILD-MDA5 Ab(+) and HC vs. DM-nonILD-MSA16(-) were the same in the cell part, intracellular part, intracellular organelle, cytoplasm, organelle part, membrane, cytoplasmic part, and nucleus; membrane-bounded organelle and intracellular membrane-bounded organelle correlated with DM-ILD-MDA5 Ab(+) exosomes when referring to CC in comparison to HC plasma exosomes. In the category of BP, cellular process, single-organism cellular process, regulation of cellular process, metabolism process, developmental processes, and biological regulation were associated with DM-ILD-MDA5 Ab(+) when compared to HC, while biosynthetic process was relevant in the other two comparisons. KEGG analysis revealed that metabolic pathway, viral infection, and oncogenesis have crucial roles in the pathogenesis of DM, while Wnt signaling and mTOR signaling were indicated to contribute to the pathogenesis in the ILD subset (Figure 6).

### 3.5. Construction of miRNA-mRNA Regulatory Network and PPI Network Analysis

**Figure 7:**
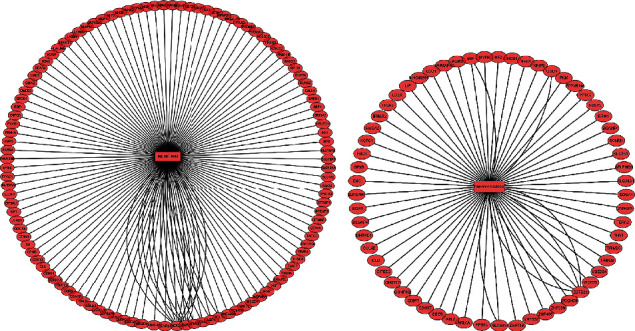
Regulatory circuits of (a) hsa-miR-1228-5p-mRNA and (b) hsa-miR-4488-mRNA. Blue circles represent miRs, and pink circles represent mRNAs. The lines represent an interaction between the nodes. The number of lines that connect genes reveals the number of case studies that have reported an association between the target genes and miRNAs according to multiMiR database. hsa: *Homo sapiens*; miR: microRNA.

**Figure 8:**
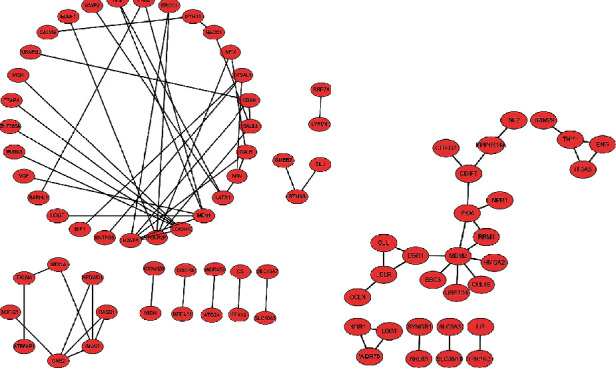
Protein-protein interaction networks for candidate target genes involved in the (a) miR-1228-5p-mRNA and (b) miR-4488-mRNA networks. Edges represent protein-protein associations. miR: microRNA.

Based on the theory of miRNA function, regulatory loops with miRNA-mRNA regulation pairs were constructed. Among the three comparisons, hsa-miR-4488 and hsa-miR-1228-5p were indicated to be the common DE miRNAs. Therefore, the intersecting miRNAs were selected to establish miRNA-mRNA regulatory networks (Figure 7) and PPI networks for CTGs (Figure 8). For the miR-4488-mRNA regulation circuit, the interaction between DExD-box helicase 39B (DDX39B) and hsa-miR-4488 indicated the strongest evidence from six experiments, followed by retinoid X receptor *β*, RNA polymerase II subunit F, menin 1, cyclin-dependent kinase inhibitor 1A, G protein subunit *β* 2, G protein subunit *α* i2, and spalt-like transcription factor 4. In addition, the PPI network based on the CTGs of miR-4488 indicated that H2A histone family member X (H2AFX) was a hub gene because it had the most nodes. In the miR-1228-5p-mRNA regulation circuit, zinc finger and BTB domain containing 22 (ZBTB22) had the strongest evidence for the interaction with miR-1228-5p, followed by solute carrier family 9 member A3, occludin, neurofibromin 2, and macrophage migration inhibitory factor, which were all evidenced twice according to multiMiR database. The PPI network for CTGs of miR-1228-5p indicated that MDM2 was a key gene because it had the most nodes.

## 4. Discussion

To the best of our knowledge, the present study was the first to report on circulating exosomes from patients with DM using a small RNA sequencing strategy. It was revealed that circulating exosome miRNA profiles had a distinct pattern between serologically defined DM subsets and HCs. Based on the circulating exosome-derived small RNA classification, eight classes of exosomal small RNA were identified: miRNA, rRNA, tRNA, snRNA, snoRNA, piRNA, Y_RNA, and unannotated small RNA. Furthermore, the DE miRNAs of plasma exosomes and their CTGs were identified. After filtering out miRNAs with low expression (RPM < 10 reads per million in circulating exosomes), using ∣log2 fold change | ≥1 and *P* < 0.01 as the cut-off threshold, several miRNAs were identified to be significantly differentially expressed between two groups. The intersecting miRNAs, hsa-miR-4488 and hsa-miR-1228-5p, were used to construct miRNA-mRNA regulatory networks.

DM is a heterogeneous group of autoimmune disorders characterized by muscle weakness, cutaneous manifestations, internal organ involvement, and paraneoplastic syndrome. Based on MSAs, occurring in idiopathic inflammatory myositis exclusively, DM may be subclassified according to their strong association with different clinical manifestations. Patients who are serologically positive for anti-MDA5 antibody usually have the acute, rapidly progressive, and potentially fatal form of ILD, which was reported to be much worse than that of antisynthetase syndromes [28]. Chest biopsies from patients with anti-MDA5 antibody indicated similar pathological findings to those for acute respiratory distress syndrome, including diffuse alveolar damage and prominent hyaline membrane deposition [29]. However, little is known about the pathogenesis of DM-ILD-MDA5 Ab(+).

Exosomes originating from various subcellular compartments are regulators of transfer of biological information locally and remotely. Exosomes are small membrane vesicles (30-150 nm in diameter) secreted by most nucleated cells [4–6]. In the present study, exosomes enriched with the reagent kit from DM patients and HCs were 121.9 ± 64.8 nm in diameter with concentrations of ~5 × 10^12^ particles/ml. The exosomal markers CD63 and CD81 were present in those particles, which was consistent with the known characteristics of exosomes.

Exosomal RNAs, which are more stable than cellular RNA, have been reported as novel biomarkers or therapeutic targets in various diseases [30, 31]. The present study demonstrated that exosome-derived miRNAs identified through NGS technology may become potential biomarkers. Among the DE miRNAs, compared to HC exosomes and DM-nonILD-MSA16(-) exosomes, 38 and 51 miRNAs were significantly upregulated, while 21 and 33 miRNAs were significantly downregulated in DM-ILD-MDA5 Ab(+) exosomes, respectively. PPI network analysis revealed that hsa-miR-4488 and hsa-miR-1228-5p may have crucial roles in the pathogenesis of DM-ILD-MDA5 Ab(+) through 112 and 60 candidate target genes, respectively. It was identified that DDX39B may possibly be a target gene of hsa-miR-4488 according to the multiMiR database. It has been reported that DDX39B interacts with the pattern recognition receptor pathway to inhibit NF-*κ*B signaling [32]. In the present study, hsa-miR-4488 was significantly upregulated in DM-ILD-MDA5 Ab(+) when compared to DM-nonILD-MSA16(-) and HC, suggesting that miR-4488 may contribute to systemic inflammation in DM-ILD-MDA5 Ab(+) by upregulating NF-*κ*B signaling through repression of DDX39B. For its part, DDX39B promotes global translation and cell proliferation through upregulating preribosomal RNA levels, thereby contributing to oncogenesis [33], similar to H2AFX as a protooncogene [34]. hsa-miR-1228-5p was upregulated in DM-ILD-MDA5 Ab(+) and was downregulated in DM-nonILD-MSA16(-) when compared to HC. Furthermore, ZBTB22 and MDM2 may be important target genes in the regulation of hsa-miR-1228-5p. MDM2, as a well-known oncogene, may also promote type 17 T-helper cell differentiation by suppressing p53-dependent transcription factor STAT5 signaling cascades, leading to activation of rheumatoid arthritis [35, 36]. However, only scarce information is available for ZBTB22. Furthermore, it was indicated that hsa-miR-4508 was a significant highly expressed miRNA in exosomes. It has been reported that circulating hsa-miR-4508 was significantly upregulated in acute myocardial infarction [37] and patients receiving dexmedetomidine [38]. However, its functions remain elusive. It was hypothesized that hsa-miR-4508 may participate in acute stress.

GO and KEGG pathway enrichment was performed on CTGs of DE exosomal miRNAs to explore the role of miRNAs packaged into exosomes from patients with DM, particularly in the ILD subset. GO analysis indicated that metabolism had a crucial role in the categories BP and CC. Several differentially regulated KEGG pathways were identified. The Wnt signaling pathway, which was indicated to be upregulated in patients with DM-associated ILD [39], and the cyclic adenosine monophosphate signaling pathway, which has been known to antagonize pulmonary fibrosis activated by phosphodiesterase 4 [40], were involved in DM-ILD-MDA5 Ab(+) exosomes when compared to both HC and DM-nonILD-MSA16(-) exosomes, suggesting that these signaling pathways may be unique to ILD, particularly in the DM-ILD-MDA5 Ab(+) subset. Transforming growth factor-*β* signaling, which was reported to be upregulated in muscle samples with sporadic inclusion body myositis [41], and mTOR signaling may be potential pathways of autoimmune diseases [42] and were included in the CTGs of DE miRNAs in the DM-nonILD-MSA16(-) exosomes, suggesting that these pathways may contribute to the pathogenesis of DM.

In conclusion, in the present study, exosomal miRNAs from plasma were profiled in two DM subsets [DM-ILD-MDA5 Ab(+) and DM-nonILD-MSA16(-)]. Selective packaging of miRNAs into exosomes, particularly hsa-miR-1228-5p and hsa-miR-4488, was observed. The results may provide promising exosomal miRNA targets for the diagnosis, prevention, and therapy of DM, particularly for the DM-ILD-MDA5 Ab(+) subset.

## 5. Limitation

This study has certain limitations. Firstly, no external data including quantitative PCR and expression matrix involved these DE miRNAs, thereby further work is warranted for the validation of hsa-miR-1228-5p and hsa-miR-4488 in DM-ILD-MDA5 Ab(+) subset. Secondly, the enrolled patients mainly came from China with little representation of the epigenetic modification in DM. In future, further work is warranted to explore the role of exosomal miRNAs in the pathogenesis of DM.

## Figures and Tables

**Table 1:** Clinical characteristics of enrolled patients.

Item	DM-ILD-MDA5 ab(+)	DM-nonILD-MSA16(-) (*n* = 5)	Healthy controls (*n* = 5)
Female sex	4 (80)	4 (80)	4 (80)
Age (years)	45.60 ± 7.49	36.40 ± 3.88	41.36 ± 3.54
Age at onset (years)	35.60 ± 8.26	41.40 ± 19.31	/
Disease duration (months)	26.00 ± 9.381	67.40 ± 37.97	/
Cutaneous features	5 (100)	3 (60)	/
Heliotrope rash	4 (80)	3 (60)	/
Gottron papules	4 (80)	0 (0)	/
Gottron sign	4 (80)	0 (0)	/
V-sign rash	1 (20)	2 (40)	/
Shawl sign	0 (0)	2 (40)	/
Cutaneous ulcers	3 (60)	0 (0)	/
Arthralgia/arthritis	3 (60)	1 (20)	/

Values are expressed as the mean ± standard deviation or *n* (%). DM-ILD-MDA5 Ab(+): DM patients with ILDs and anti-melanoma differentiation-associated protein 5 antibody-positive status; DM-nonILD-MSA16(-): DM patients without ILDs who were negative for 16 detectable myositis-specific antibodies; ILD: interstitial lung disease; DM: dermatomyositis.

## Data Availability

The datasets used and/or analyzed during the current study are available from the corresponding author on reasonable request.

## Supplementary Materials

Supplementary Materials Supplementary Materials Table S1. All differentially expressed miRNAs in HC versus DM-ILD-MDA5 Ab (+). Table S2. All differentially expressed miRNAs in DM-nonILD-MSA16 (-) versus DM-ILD-MDA5 Ab (+). Table S3. All differentially expressed miRNAs in HC versus DM-nonILD-MSA16 (-).
